# The Influence of Different Metal Ions on the Absorption Properties of Nano-Nickel Zinc Ferrite

**DOI:** 10.3390/ma11040590

**Published:** 2018-04-11

**Authors:** Zhijun Ma, Changye Mang, Xingyuan Weng, Qi Zhang, Liwei Si, Haitao Zhao

**Affiliations:** College of Mining, Liaoning Technical University, Fuxin 123000, China; mcy0515@163.com (C.M.); wengxingyuan2008@163.com (X.W.); zhangqi8891@163.com (Q.Z.); sogoho@126.com (L.S.); haitaozhao0718@126.com (H.Z.)

**Keywords:** hydrothermal method, ferrite, microwave absorbing properties

## Abstract

The hydrothermal method was used to dope different amounts of Co^2+^, Mn^2+^, and Cu^2+^ in nano-nickel zinc ferrite powder. X-ray diffraction (XRD), a scanning electron microscopy (TEM), and a vector network analyzer (VNA) were used to explore the influence of doping on particle size, morphology, and electromagnetic wave absorption performance. Pure nanometer cobalt nickel zinc ferrite phase was prepared using the hydrothermal method with an increasing Co^2+^ content. Results showed that the grain type structure changed from a spherical structure to an irregular quadrilateral structure with the average particle size increasing from 35 nm to 60 nm. The lattice constant increased from 0.8352 to 0.8404 nm with Co^2+^ doping. The increasing Co^2+^ can change the position of the absorption peak, increase the bandwidth of the absorber, and improve the performance of the materials in GHz low frequency. The doping ratio of Mn^2+^ can affect the size of the lattice constant, but nanocrystals are easy to reunite without improving the electromagnetic loss. However, the absorbance performance decreases. For the doping of Cu^2+^, there is an agglomeration phenomenon. When the doping quantity is 0.15, the absorbing wave performance becomes better.

## 1. Introduction

Nano nickel zinc ferrite is a kind of ruby structure of soft magnetic materials. It exhibits excellent absorbing performance due to its high resistivity, high permeability, low dielectric loss, and low temperature coefficient [1,2]. It also plays an important role as an electromagnetic material in high frequency inductive magnetic cores, magnetic recording materials, cable or wireless communications, radio, television, radar, and stealth materials. It has an additional benefit of being a low cost material [3,4].

In the unit cell of spinel ferrite, there are many vacancies produced by an ion valence balance between action and the absence of space, which makes it easy to use another metal as a dopant. Ferrite magnetic loss and changes in absorption performance can be realized by doping metal ions with different electromagnetic wave absorption strengths, frequency band widths, and electromagnetic parameters of controlled regulation [5,6]. However, introduction of a dopant does not necessarily improve the performance of the material. Therefore, it is important to study the effect of doping different ions and mixtures in ferrite.

Ramesh S. studied ferrite doped with metal nitrate citric acid ester-based matrix prepared by Ni-Zn ferrite nano materials with sol gel combustion produced by Co^2+^ or Mn^2+^ and found that it exhibited wave-absorbing properties in the low-frequency phase [7]. A study of ferrites doping by Zhao et al. showed that the dry gel formed by metal nitrates and citric acids can be used to synthesize pure phase ferrite with an average particle size of about 60 mm through a self-burning process [8]. Xiong et al. studied the electromagnetic parameters of six angular ferrites doped with different ions [9]. Using different Mn-Zn ferrite volume integral methods, Adriana M. Gama explored the complex permeability of rubber radar absorbing materials and analyzed the dielectric permittivity [10]. T.J. Shinde studied the properties of nickel zinc ferrite including saturation magnetization, magnetic exchange polarization, and cation distribution [11]. In this paper, the crystal shape and microwave absorbing properties of the samples were compared using the hydrothermal method.

It is difficult for single ferrite absorbing materials to meet the requirements for a good wave absorbing material, which include light absorbing, thin, high quality, wide frequency range, and strong absorption. Therefore, it is necessary to mix ferrite with other absorbents to use the synergistic effect of composite materials as well as the different absorption bands and the absorption mechanisms of multivariate composite materials in order to achieve a better absorbing material. In recent years, the development of composite absorbent materials has been the focus of research in the field of absorbing wave materials [12].

## 2. Experimental

### 2.1. Experimental Reagent

All the chemical reagents including Fe(NO_3_)_3_·9H_2_O; Zn(NO_3_)_2_·6H_2_O; Ni(NO_3_)_2_·6H_2_O; Co(NO_3_)_2_·6H_2_O, MnCl_2_·4H_2_O, CuSO_4_·5H_2_O, NaOH, and CH_3_CH_2_OH are analytical pure (AR); PEG(H(OCH_2_CH_2_)_n_OH), CP; distilled water(H_2_O) was prepared by the Mineral processing engineering laboratory.

### 2.2. Experimental Method

The amounts of ferric/nickel/zinc nitric acid, cobalt/chlorinated manganese/copper sulphate, and polyethylene glycol varied in each experiment.

The above raw materials were dissolved in 150 mL of distilled water and polyethylene glycol (PEG) was added to the mixture. The mixture underwent 30 min of ultrasonic processing to obtain an evenly dispersed solution. The solution was transferred to a flask and then into a water bath where the temperature was held at a constant 40 °C. The solution was stirred at a rate of 180 r/min for 1 h. During stirring, one drop of 2 mol/L NaOH was added to the mixture every two seconds. This allowed the pH to be adjusted to 10 to generate the reaction precursors.

After 8 h of aging at a normal temperature, the supernatant was removed. The lower layer contained the reaction precursor. This was shaken in a stainless steel thermal water kettle. 

The water thermal response was monitored at 180 °C for 8 h in the crystallization reaction kettle. Afterward, a value of 80% was obtained. After the reaction, the material was successively washed with distilled water, which was followed by four washes with anhydrous ethanol. The material was then vacuum suctioned and filtered at 80 °C in a thermostatic drum drying oven. The product was then ground into a powder to yield the nanometer ferrite.

### 2.3. Test Sample Representation

A BRUKER D8 ADVANCE X-ray Diffractometer was used to analyze the compositional phases of the samples. The measurement conditions for these experiments include the following: b Cu K alpha wavelength of 1.5406 Å, 40 kV voltage, tube current of 30 mA, scanning speed of 10°/min, and scanning range of 25~65°. With the Scherrer formula [12], it is possible to estimate the grain size of the sample particles (D = kλ/βcosθ, k = 0.89, λ = 0.154056 nm, β is half high width of peak, and θ is diffraction angle). Using a JEOL JEM-2010 transmission electron microscope (TEM), the crystal morphology was analyzed using the values in which the microstructure and particle size resolution was 0.1~0.2 nm and the accelerating voltage was 200 kV. The samples used for electromagnetic measurements were prepared by homogeneously mixing the hybrid materials with wax (the weight ratio of the prepared powder was about 30%) and then the mixture was pressed into a toroid with an outer diameter of 7.0 mm, an inner diameter of 3.0 mm, and a thickness of 2.0 mm. Using an AGILENT HP8722ES vector network analyzer (VNA), a coaxial-line method was undertaken to examine the samples’ dielectric constant and permeability between 1 and 12 GHz frequencies. With the electromagnetic parameters of absorbing materials (*μ_r_*, *ε_r_*), the instrument parameters and the test results MATLAB were used to calculate the corresponding absorbing reflectivity and the relationship with the frequency curve. On the basis of the measured data of permittivity and permeability, reflection loss (*RL*) usually can be calculated by using the equation below.

$$RL\left(\mathrm{dB}\right)=20\log_{10}|\frac{Z_{in}-1}{Z_{in}+1}|$$

where *Z_in_* is the input characteristic impedance, which can be expressed as the equation below.

$$Z_{in}=\sqrt{\frac{\mu_{r}}{\varepsilon_{r}}}\tanh\left[j\left(\frac{2\pi fd}{c}\right)\sqrt{\varepsilon_{r}\mu_{r}}\right]$$

where *ε_r_* and *μ_r_* are the complex permittivity and permeability of the absorber, respectively, *f* is the frequency, *d* is the thickness of the absorbent, and *c* is the velocity of light in free space.

The Loss factor can be calculated by using the following equation.

$$\tan\delta =\tan\delta_{e}+\tan\delta_{m}$$

where tan*δ* is the loss factor, tan*δ*_e_ is the dielectric loss ($\tan\delta_{e}=\varepsilon \prime \prime /\varepsilon \prime$), and tan*δ*_m_ is the magnetic loss ($\tan\delta_{m}=\mu \prime \prime /\mu \prime$).

## 3. Results and Discussion

### 3.1. The Influence of Doped Co^2+^ Content on the Absorbing-Wave Performance of Nano-Nickel Zinc Ferrite

**Figure 1:**
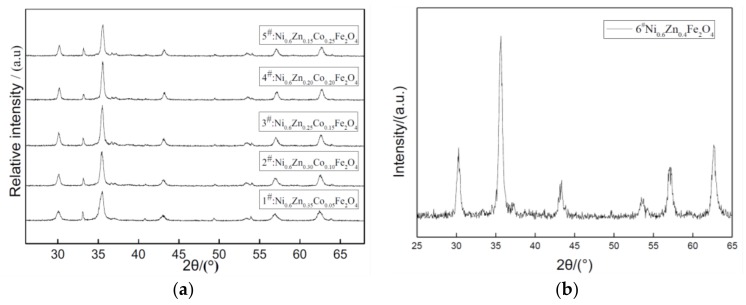
X-ray diffraction graph patterns of samples prepared with different Co^2+^ content (**a**) and without Co^2+^ content (**b**).

The XRD patterns of nano Ni_0.6_Zn_(0.4−x)_Co_x_Fe_2_O_4_ for several samples with varying Co^2+^ content are shown in Figure 1. For all the samples’ X-ray diffraction patterns, peak positions correspond with the nickel zinc ferrite (111) crystal plane, (220) face, (311), (400), (422), (511), and (440) faces. The cubic crystal system of the pure spinel structure is represented without the presence of any impurity phases such as seen in alpha Fe_2_O_3_. This indicates that an experimental synthesis of the pure cobalt nickel zinc ferrite phase is possible, but it is difficult to achieve for the dry method of doping ions. When the Co^2+^ content increases, the diffraction peak intensity decreases first. However, when Co^2+^ doping reaches x = 0.15, no missing diffraction peaks are seen, which indicates that the crystallization is complete. The intensity of the (311) Bragg reflection is strong, which suggests that the preferred crystal crystallization orientation is (311). Additionally, the peak is particularly wide, which indicates that the polycrystalline material is nano-sized [13,14]. In applying the Scherrer formula, the average grain size in these cobalt-doped nickel zinc ferrites and the average particle size of the crystal grain increases by 35~60 nm with doping while the lattice constant increases from 0.8352 nm to 0.8404 nm in Table 1. The hydrothermal method of doping can allow for the introduction of large elements in the Co^2+^ lattice, which decreases the solution surface tension. This results in a reduction in the formation of the new phase activation energy. It also avoids the defects of the traditional method, which may promote dopant and ferrite mixing, and also controls the specific amount of dopant in the nanomaterials to maintain a certain stoichiometric ratio.

**Figure 2:**
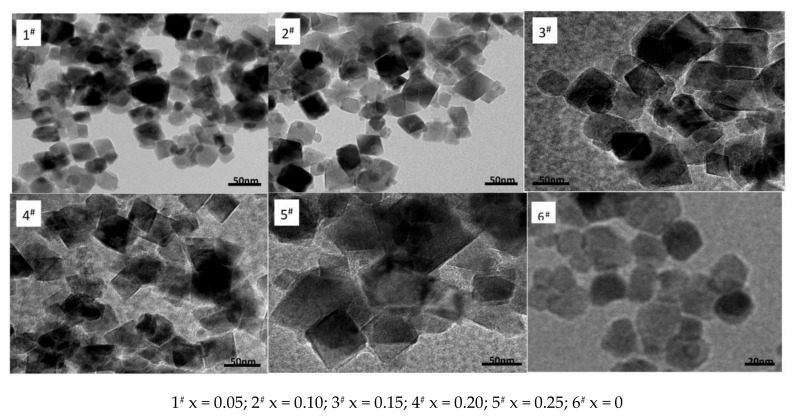
Transmission electron micrograph of samples prepared with different Co^2+^ content.

Figure 2 shows nano Ni_0.6_Zn_(0.4−x)_Co_x_Fe_2_O_4_ TEM micrographs. In the figure, it can be observed that after doping with Co^2+^, the intrinsically spherical nickel zinc ferrite nanoparticles assumed a more irregular quadrilateral structure. With increasing Co^2+^ content, the grain size increases, the irregular grain arrangement becomes denser, it exhibits poorer dispersion, and the grain becomes more uneven in size. The average particle size reaches 60 nm, which is roughly the same size as the one obtained with the Scherrer formula in the XRD results. Doping with Co^2+^ nanometer nickel zinc ferrite can visibly change the direction of the crystal growth. This can affect the samples’ surface tension and surface energy. The radius of doped Co^2+^ is 453 (pm), which is smaller than the radius of Ni^2+^ or Zn^2+^. It is possible that it occupies the oxygen ions’ tetrahedral hole in a very minute amount, if at all. Priority is taken to enter the octahedra (B) in which part of the Fe^3+^ ions are removed [15]. The lattice distortion causes distortion of the spinel structure and the grain shape becomes irregular. Additionally, as the Co^2+^ content increases, the Co^2+^ that does not enter the crystal structure is dispersed to the grain boundary in which the grain grows significantly larger.

A microwave passing through a medium undergoes three main mechanisms including reflection, absorption, and penetration. As the microwave passes through a glossy dispersive material, its reflection is affected by factors such as complex permittivity, magnetic permeability, sample thickness, and specific surface area. Real permittivity and permeability relate to the amount of polarization in the materials and indicate the ability to store electromagnetic energy. Imaginary permittivity and permeability signify magnetic loss and energy dissipation within a material, which result from conductance, resonance, and relaxation.

**Figure 3:**
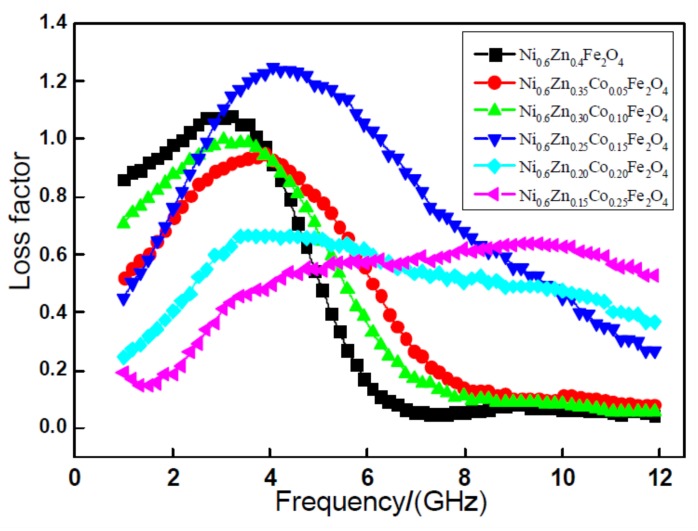
Curves of tan*δ* and frequency for the Ni_0.6_Zn_(0.4−x)_Co_x_Fe_2_O_4_ sample.

Figure 3 exhibits the relationship curve of the tangent delta with frequency variation of the loss factor tangent delta of the nano Ni_0.6_Zn_(0.4−x)_Co_x_Fe_2_O_4_. As the graph shows, with an increase in frequency, the nano Ni_0.6_Zn_(0.4−x)_Co_x_Fe_2_O_4_ system of the tan delta value exhibits an overall trend of increasing after first decreasing. However, with an increase in the Co^2+^ dopant and the change of loss factor, tan delta is nonlinear. When doping x < 0.15, the tangent of the tangent of the tangent exhibits an increase and, when the quantity is small, the tangent delta is smaller than that of the un-doped parent material. When the dopant amount reaches x = 0.15, the material loss factor of the strongest peak moves to higher frequency. The peak at 1.25 is significantly more intense than that of other samples. At the same time, to achieve the most effective frequency width, the wave absorption performance is optimal here. When the doping quantity is x = 0.20, 0.25, the effective frequency band widens, but the tangent delta grows smaller and the absorbing effect is not very satisfactory [16]. A comprehensive analysis of the electromagnetic loss shows that within the 1~12 GHz frequencies, when the Co^2+^ doping amount is 0.15 of the nano Ni_0.6_Zn_0.25_Co_0.15_Fe_2_O_4_, it exhibits an enhanced performance of the electromagnetic wave and electromagnetic loss in which the wave absorption performance is better.

This is because the nanometer Ni_0.6_Zn_(0.4−x)_Co_x_Fe_2_O_4_, Ni^2+^, and Co^2+^ prefer to occupy the octahedra (B). With an increase in the Co^2+^ content, the grain boundary phase reduces. Demagnetization can decrease this grain boundary growth and increase the strength of Ni_0.6_Zn_(0.4−x)_Co_x_Fe_2_O_4_, which improves the magnetic hysteresis loss of the material. At the same time, with the octahedral crystal lattice distortion (B), the internal stress increases. With less material containing Fe^2+^, the Fe^2+^-Fe^3+^ electron mobility is reduced. This makes the hole type more conductive and the dielectric loss increase. With an increase in Co^2+^ content, the octahedral Co^3+^ (B), the Co^3+^ undergoing electron exchange with Co^2+^, and the octahedron (B) Fe^2+^ and Fe^3+^ electronic exchange disappear. As a result, the equivalent electronic exchange decreases and the electrical conductivity is abated. The dielectric constant and loss are also reduced. In this manner, in the nano nickel zinc ferrite, the Co^2+^ dopant can improve the electromagnetic loss properties of the nickel zinc ferrite nanoparticles and can effectively broaden the spectrum. When the doping amount reaches x = 0.15, the electromagnetic loss characteristics of the sample are the most positive in which the absorbing effect is the greatest.

**Figure 4:**
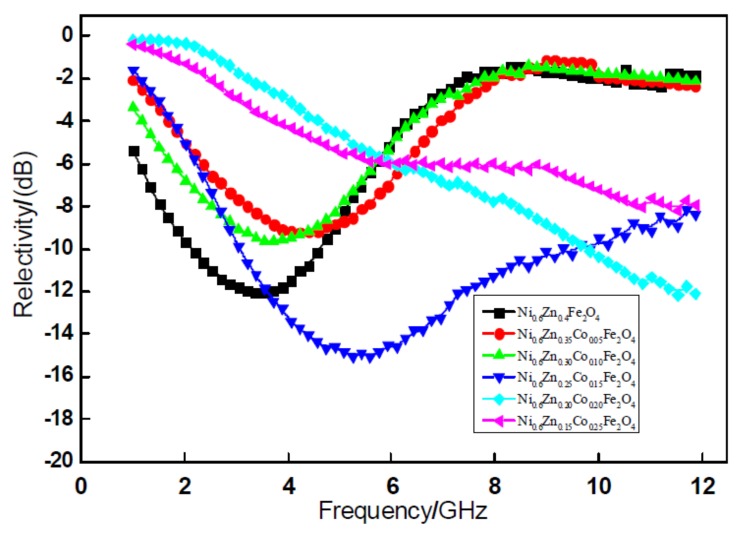
Curves of reflectivity and frequency for the Ni_0.6_Zn_(0.4−x)_Co_x_Fe_2_O_4_ sample.

Figure 4 shows the relationship curve of the absorbency reflectivity of 2-mm nanometer Ni_0.6_Zn_(0.4−x)_Co_x_Fe_2_O_4_. The figure reveals that, within the 1~12 GHz band, the nano nickel zinc ferrite absorbing reflectivity is 12.02 dB, which is valid for the 1~6 GHz frequency band. After doping Co^2+^, along with an increase in the dopant x, when x = 0.05, 0.10, the absorbing peak reflectivity is 9.21 dB and 9.64 dB. When the Co^2+^ doping quantity is low, the material’s absorbing effect is not clear. When the doping quantity x = 0.15, the absorbing wave reflectivity is reduced from −12.01 dB to −15.05 dB. The effective band width nearly doubles, but the absorbing wave performance is the highest value. Although an excessive amount of Co^2+^ doping increased the reflectivity, there was no clear peak value. Even though peaks tend to move to higher frequency, within the test frequency band, the overall wave absorption bands improved significantly. At the same time, by doping Co^2+^, the absorption peak position can change [17]. The peak value of the curve in the diagram and the corresponding frequency are shown in Figure 3. In this regard, the appropriate amount of Co^2+^ can effectively be used to increase the absorbance frequency and improve the absorbing performance of the material. Doping Co^2+^ ions in the ferrite use the compensation of negative anisotropy to achieve a stable domain wall and increase the electromagnetic loss. These factors meet the requirements of electromagnetic wave ferrite devices.

### 3.2. The Influence of Mn^2+^ Content on the Absorption Properties of Nano-Nickel Zinc Ferrite

**Figure 5:**
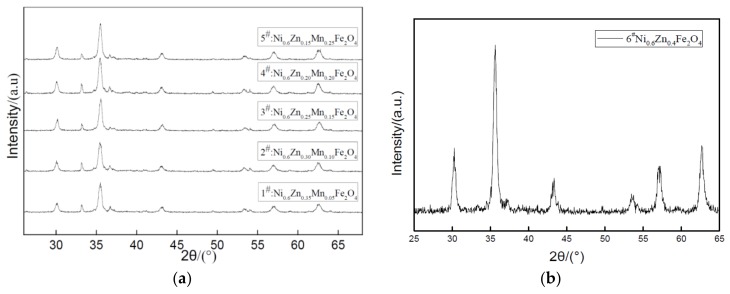
X-ray diffraction graph patterns of samples prepared with different Mn^2+^ content (**a**) and without Mn^2+^ content (**b**).

Figure 5 shows the XRD pattern of nano Ni_0.6_Zn_(0.4−x)_Mn_x_Fe_2_O_4_ mixing different levels of Mn^2+^. Table 2 exhibits the ferrite component and structure parameters with various Mn^2+^ content. As can be seen from Figure 5 and Table 2, all the samples, in compliance with the standard atlas JCPDS reference, exist in the single phase spinel structure. No other impurities can be detected and the five main diffraction peaks appear in the sample at 35°. The diffraction peak intensity increases as the amount of Mn^2+^ doped increases from 346.33 to 574.67. The peak intensity significantly sharpens with the dopant, which indicates that doping Mn^2+^ into the crystal lattice does not change the crystal structure. However, it increases its crystallinity and promotes the crystallization of pure phase nanometer nickel manganese zinc ferrite. With an increase in x, the (311) crystal plane diffraction Lord peak moves to smaller angles. This is associated with an increase in the lattice constant of the product [18]. The grain size at the same time also experiences a slight increase, but this increase is less than that of the cobalt nickel zinc ferrite nanoparticles. This is because the Ni_0.6_Zn_(0.4−x)_Co_x_Fe_2_O_4_ ionic radius is larger than that of Mn^2+^. The dopant may enter the tetrahedron to replace the original small Zn^2+^ ion radius and Fe^3+^. Due to the volume of A, the crystal structure effect makes the lattice constant change by increasing it from 0.8352 nm to 0.8440 nm. But the lattice constant decreases when the doping quantity x = 0.15, so the doping ratio of Mn^2+^ affects the size of the lattice constant.

**Figure 6:**
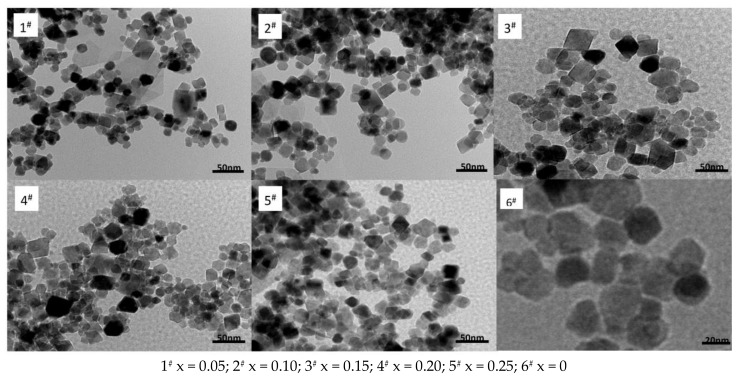
Transmission electron micrograph of samples prepared with different Mn^2+^ content.

Figure 6 reveals a nano Ni_0.6_Zn_(0.4−x)_Mn_x_Fe_2_O_4_ TEM image. The image at the nanometer scale shows that the nickel and manganese zinc ferrite grain size is spherical in nature. There are fewer quadrilateral, loose aggregate surfactant, and large translucent, irregular particle shapes that may form in the reaction process. The fewer Mn^2+^ content, the smaller the ferrite particles are with most being roughly 30 nm in size, which is consistent with the XRD results. The grain growth is good and evenly dispersed. However, with an increase in Mn^2+^ content, the grain grows. With this growth, the particles assume looser, aggregate quadrilateral structures and are relatively dense, uneven in size, and have a mean grain size of 40 nm. They also exhibit poor dispersion and agglomeration. Because of the hydrothermal preparation of nickel and manganese zinc ferrite nano powder, the nanometer grain specific surface area is larger [19]. Due to the influence of the surface effect and the magnetic attraction between the particles and the molecular inter-atomic forces, the particles reunite easily into large aggregates at the interface. This works to reduce the surface and static magnetic energy. At the same time, the grain growth speed is not easy to control and the sizes will differ. The second is capillary contraction in the drying process, which causes the agglomeration.

**Figure 7:**
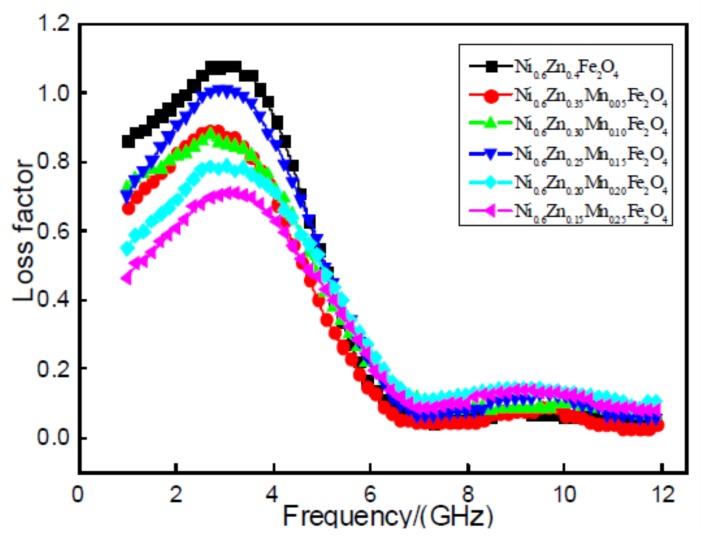
Curves of tanδ and frequency for the Ni_0.6_Zn_(0.4−x)_Mn_x_Fe_2_O_4_ sample.

Figure 7 shows the loss factor tan delta of nano Ni_0.6_Zn_(0.4−x)_Mn_x_Fe_2_O_4_. It does not improve after doping with Mn^2+^. The nano nickel zinc manganese ferrite magnetic loss, loss factor, tan delta value in the 1~12 GHz frequency, and the increase of frequency first increased after decreasing. With the increase of Mn^2+^ dopant, the loss factor, tan*δ* value, does not exhibit a linear change, but is reduced after increasing and then decreases once again. When the doping quantity is x = 0.05, 0.10, the tan*δ* value does not change significantly. When the doping amount is x = 0.15, in the 1~6 GHz frequency, it is bigger than the loss factor in the other doped samples. The effective frequency band widens slightly, but is still below the doping tan and the size of the tan*δ* value. When the doping quantity x = 0.20, 0.25, the tan*δ* value decreases with an increase in doped Mn^2+^. The excess Mn^2+^ dopant minimizes the dissipation factor. Doping of Mn^2+^ in the nano nickel zinc manganese ferrite shows a loss in magnetic performance and does not reach the effect of the wave absorption performance of the reinforced material. This is because the Mn^2+^ effective magnetic moment is less than that of Fe^3+^. With the increase in Mn^2+^ dopant, the Fe^3+^ concentration is reduced, which results in a decrease in the effective magnetic moment of the nanometer nickel manganese zinc ferrite and the saturation magnetization. This leads to loss in the overall electromagnetic intensity [20].

**Figure 8:**
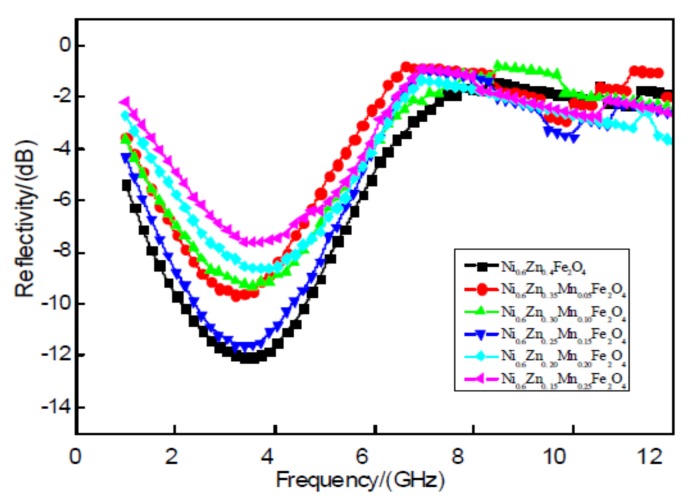
Curves of reflectivity and frequency for the Ni_0.6_Zn_(0.4−x)_Mn_x_Fe_2_O_4_ sample.

Figure 8 shows 2 mm nanoscale Ni_0.6_Zn_(0.4−x)_Mn_x_Fe_2_O_4_. In the diagram, between the 1~7 GHz band, the nano nickel zinc manganese ferrite absorbing peak reflectivity is relative to the electromagnetic wave absorbing effect at low frequency. With the increase of Mn^2+^ doping amount, the absorbing peak reflectivity of the sample is: 9.65 dB, 9.31 dB, 11.65 dB, 8.64 dB, and 7.63 dB. The absorbing reflectivity increased by 12.01 dB at the doping amount of x = 0.25–7.63 dB, which is shown by the curve in Figure 8. As a result, the Mn^2+^ impurity in the nano-nickel-zinc ferrite decreases the absorbing-wave performance of the material. With this decrease in the absorbing effect, the effective band width will not change significantly. This is because when doping with Mn^2+^, the octahedral Fe^3+^ (B), the concentration of Fe^2+^, and the octahedron (B) Fe^2+^ with its positive magnetocrystalline anisotropy constant are replaced. The Mn^2+^ negative magnetocrystalline anisotropy constant makes it smaller and, therefore, reduces the electromagnetic loss of the material. This weakens the wave absorption performance.

### 3.3. The Influence of the Doped Cu^2+^ Content on the Performance of Nano-Nickel Zinc Ferrite Absorbability

**Figure 9:**
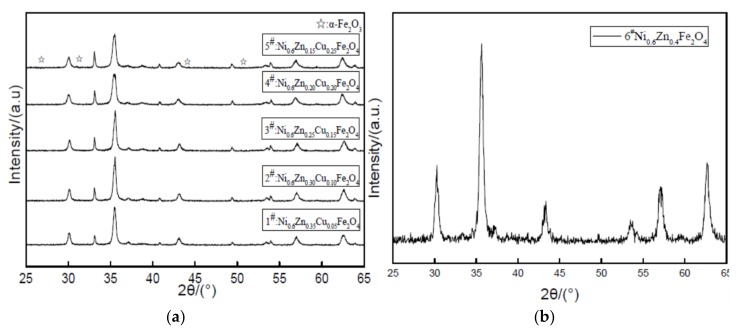
X-ray diffraction graph patterns of samples prepared with different Cu^2+^ content (**a**) and without Cu^2+^ content (**b**).

As can be seen from the Figure 9, five samples of peak type are compared with standard atlas JCPDS and all have the characteristic diffraction peaks of the spinel ferrite. The main diffraction peak position essentially remains unchanged. With an increase in the Cu^2+^ dopant, the intensity of the main (311) peak at 718.00 and decreases to 496.67. The diffraction peak gradually broadens. While the crystalline degree is smaller, there are different alpha Fe_2_O_3_ diffraction peak intensities in the pattern. The nanometer copper nickel zinc ferrite peaks are impurities. Table 3 shows the copper nickel zinc ferrite grain sizes. The lattice constant increased with an increase in Cu^2+^ dopant while the grain size and lattice constant undergo an opposite change. Because of the copper defects in the lattice, the nickel-zinc ferrite lattice distortion is not conducive to the growth of the lattice constant [21]. The grain size is smaller because the Cu^2+^ first entered the lattice on the surface of the grain. The surface defects of the grain prevent the combination between grains. This inhibits grain growth. Conversely, due to the contraction of the cell size, the Cu^2+^ radius is smaller than the radii of Ni^2+^ and Zn^2+^, which causes lattice distortion of the nanocrystals and results in a decrease in the cell size. As revealed here, the doped influence of Cu^2+^ on the structural parameters of the ferrite will affect the material’s absorbability.

**Figure 10:**
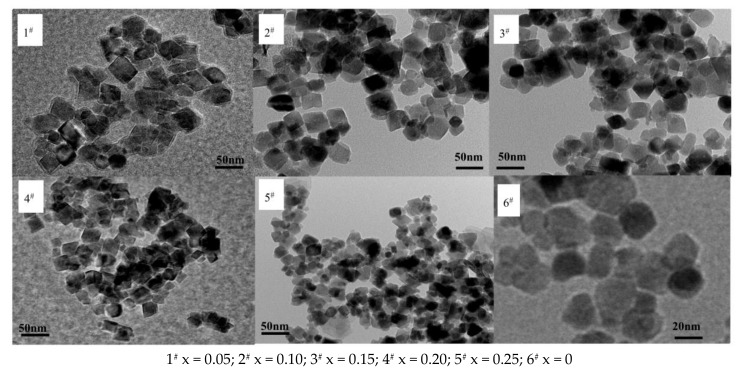
Transmission electron micrograph of samples prepared with different Cu^2+^ content.

Figure 10 exhibits a nano Ni_0.6_Zn_(0.4−x)_Cu_x_Fe_2_O_4_ TEM diagram. As shown in the figure, the generated nano nickel zinc ferrite morphology has characteristics of the copper quadrilateral structure, grain shape, and size distribution. When the Cu^2+^ content decreases, the ferrite particles are larger than the unadulterated ones, but the spatial dispersion is still relatively good with decent agglomeration of the material. With an increase in Cu^2+^ dopant, the grain size is smaller and the agglomeration phenomenon is more dramatic. When the Cu^2+^ dopant reaches x = 0.25, the nanometer copper nickel zinc ferrite grain size changes from 59.63 nm to 32.46 nm and the agglomeration phenomenon intensifies. This is because the radius of Cu^2+^ and Ni^2+^ are similar. In the reaction, Cu^2+^ fills the space between the mutual ions and maintains the balance of charge. Through a spin mechanism, it inhibits the growth of particles. With the increase in the Cu^2+^ dopant, the grain size decreases. At the same time, transmission electron microscopy (TEM) sample preparation takes roughly half an hour. Through the ultrasonic oscillations of the scattered material, the sample structure is not damaged, which indicates that the structure itself is very stable.

**Figure 11:**
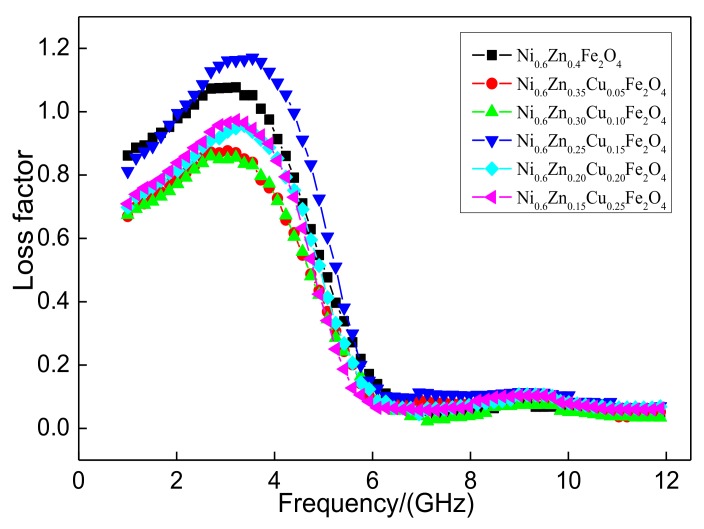
Curves of tanδ and frequency for the Ni_0.6_Zn_(0.4−x)_Cu_x_Fe_2_O_4_ sample.

Figure 11 shows the loss factor tan*δ* of the nanometer Ni_0.6_Zn_(0.4−x)_Cu_x_Fe_2_O_4_. The loss factor is overall consistent while the change in frequency first decreases and then increases. There are no special fluctuations, which illustrates that the Cu^2+^ doping does not affect the effective frequency band range of the nanometer nickel zinc ferrite magnetic loss in the 1~5 GHz frequency range. With an increase in Cu^2+^ dopant, the loss factor or tanδ value decreases after a quick increase and then decreases again. When the Cu^2+^ content is too low, the tangent essentially goes unchanged. When the doping quantity reaches x = 0.15, the loss factor of the material, “the tangent of the tangent of the tangent,” exhibits a large change, which reaches a maximum of 1.17. This is where the absorbance-wave performance is at the highest value. When the Cu^2+^ content is too high, the tangent of the tangent is smaller and much less than the unadulterated value. As revealed by a comprehensive analysis of the electromagnetic loss, within the 1~12 GHz band, Cu^2+^ doping did not improve the absorbance area. The appropriate Cu^2+^ doping amount is necessary to reach the absorbance performance of the reinforced material. When the Cu^2+^ dose reaches 0.15, the nanometer Ni_0.6_Zn_0.25_Cu_0.15_Fe_2_O_4_ is more suitable for electromagnetic dissipation of the electromagnetic waves and, therefore, it performs better.

**Figure 12:**
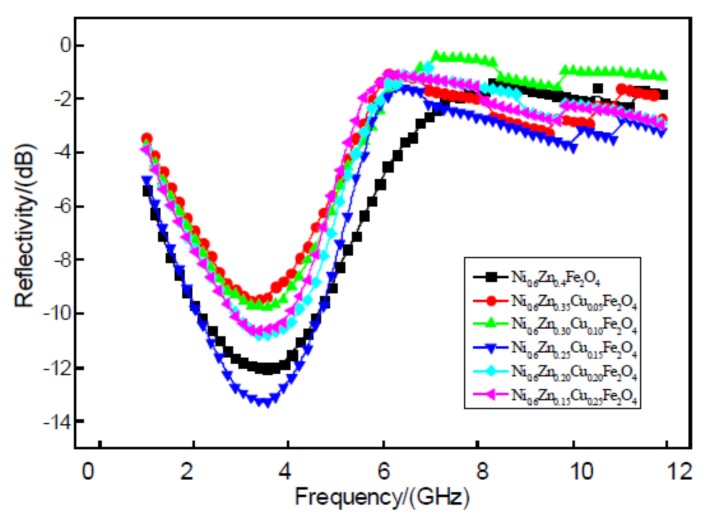
Curves of reflectivity and frequency for the Ni_0.6_Zn_(0.4−x)_Cu_x_Fe_2_O_4_ sample.

Figure 12 displays a 2 mm nanoscale image of the Ni_0.6_Zn_(0.4−x)_Cu_x_Fe_2_O_4_ material. Within the test range, six samples showed a strong reflectivity absorption peak at 1~5 GHz frequencies while the other peaks were not as intense. With the addition of Cu^2+^, the position of the strong peak does not change and the wave band does not widen but instead exhibits a slight narrowing. The Cu^2+^ doping amount at x > 0.15 reveals a greater influence on the reflectivity of the samples. Such reflectivity values of −10 dB are obtained near the 3.5 GHz absorbing peak reflectivity from 12.01 dB down to a dopant concentration of x = 0.15, −13.29 dB. While the absorbance performance improves, the other doping amount of the reflectivity is greater than 12.01 dB. This overall weakens the wave absorption performance of the nanometer nickel zinc ferrite. This may be due to the material’s structure. Zn^2+^ first enters the tetrahedral holes (A) while Cu^2+^ and Ni^2+^ prefer to occupy the octahedral holes (B). At the same time, Fe^3+^ occupies both A and B [22]. The magnetic moment of Fe^3+^ is 5 μB. The magnetic moment of Cu^2+^ (2 μB) is greater than that of Zn^2+^, which carries no magnetic moment. Because some Cu^2+^ ions replace A and Zn^2+^, the B net magnetic moment is reduced, which decreases the magnetic loss and weakens the wave absorption performance. However, just the right amount of Cu^2+^ could enter the tetrahedral holes by arriving at the location of Fe^3+^ A. The B to B A net magnetic moment could increase, enhance the A magnetic moment of exchange, and, thereby, increase the magnetic loss. This would then improve wave absorption performance. Therefore, it is possible to apply the appropriate amount of Cu^2+^ to enhance the absorbing performance of the material.

## 4. Conclusions

Nanometer manganese cobalt/nickel zinc/copper ferrite was successfully prepared under the following conditions: the surfactant polyethylene glycol was added to a solution to obtain a pH value of 10 in which the Ni^2+^ content was 60%, the iron concentration was 0.33 mol/L, and the crystallization temperature was 180 °C. The solution then underwent hydrothermal crystallization in 8 h with Co^2+^, Mn^2+^, and Cu^2+^.

The material was doped with pure Co^2+^ prepared nano cobalt nickel zinc ferrite. When the Co^2+^ dopant increased, the original spherical-like grain assumed an irregular quadrilateral structure and the average particle size increased from 35 nm to 60 nm. For Ni_0.6_Zn_0.25_Co_0.15_Fe_2_O_4_, the reflectivity absorbance changed from 12.01 dB to 15.05 dB and the absorbance spectrum width nearly doubled. This resulted in an overall improvement in the material’s gigahertz frequency wave absorption performance. Compared to the un-doped material, the electromagnetic loss performance overall decreased and the absorbance wave reflectivity increased while the absorbance effect decreased. There was no profound effect on the doping of Cu^2+^ nano nickel zinc ferrite on the absorbance spectrum of the electromagnetic loss at 3.5 GHz. Doping of Mn^2+^ in the nano nickel zinc manganese ferrite does not reach the effect of the wave absorption performance in the reinforced material. Ni_0.6_Zn_0.25_Cu_0.15_Fe_2_O_4_ reached a maximum loss factor of 1.17 and an absorbance reflectivity of 13.29 dB. This material revealed the best wave absorption performance while the other doped analogs in the series did not attain the effect of wave absorption performance in the reinforced material.

## Figures and Tables

**Table 1:** Composition and structure parameters of ferrite.

Structural Formula	2θ/(°)	Lattice Parameter	(311) Priority Crystallization Diffraction Peak		
			FWHM/rad	Intensity/a.u.	Size/nm
Ni_0.6_Zn_0.4_Fe_2_O_4_	35.62	0.8352	0.3828	468.56	20.53
Ni_0.6_Zn_0.35_Co_0.05_Fe_2_O_4_	35.53	0.8360	0.2362	411.33	34.94
Ni_0.6_Zn_0.30_Co_0.10_Fe_2_O_4_	35.49	0.8362	0.1968	460.33	41.93
Ni_0.6_Zn_0.25_Co_0.15_Fe_2_O_4_	35.44	0.8373	0.1680	555.33	49.12
Ni_0.6_Zn_0.20_Co_0.20_Fe_2_O_4_	35.58	0.8381	0.1574	499.67	52.44
Ni_0.6_Zn_0.15_Co_0.25_Fe_2_O_4_	35.34	0.8404	0.1378	487.33	59.86

**Table 2:** Composition and structure parameters of ferrite.

Structural Formula	2θ/(°)	Lattice Parameter	(311) Priority Crystallization Diffraction Peak		
			FWHM/rad	Intensity/a.u.	Size/nm
Ni_0.6_Zn_0.4_Fe_2_O_4_	35.62	0.8352	0.3828	468.56	20.53
Ni_0.6_Zn_0.35_Mn_0.05_Fe_2_O_4_	35.61	0.8354	0.3023	346.33	27.31
Ni_0.6_Zn_0.30_Mn_0.10_Fe_2_O_4_	35.58	0.8362	0.2833	360.33	29.14
Ni_0.6_Zn_0.25_Mn_0.15_Fe_2_O_4_	35.24	0.8382	0.2520	488.00	32.72
Ni_0.6_Zn_0.20_Mn_0.20_Fe_2_O_4_	35.49	0.8440	0.2475	571.67	33.34
Ni_0.6_Zn_0.15_Mn_0.25_Fe_2_O_4_	35.44	0.8395	0.2046	574.67	40.34

**Table 3:** Composition and structure parameters of ferrite.

Structural Formula	2θ/(°)	Lattice Parameter	(311) Priority Crystallization Diffraction Peak		
			FWHM/rad	Intensity/a.u.	Size/nm
Ni_0.6_Zn_0.4_Fe_2_O_4_	35.62	0.8352	0.3828	468.56	20.53
Ni_0.6_Zn_0.35_Cu_0.05_Fe_2_O_4_	35.51	0.8365	0.1384	658.67	59.63
Ni_0.6_Zn_0.30_Cu_0.10_Fe_2_O_4_	35.51	0.8370	0.1578	718.00	52.30
Ni_0.6_Zn_0.25_Cu_0.15_Fe_2_O_4_	35.56	0.8380	0.1774	654.33	46.53
Ni_0.6_Zn_0.20_Cu_0.20_Fe_2_O_4_	35.54	0.8380	0.1971	532.67	41.87
Ni_0.6_Zn_0.15_Cu_0.25_Fe_2_O_4_	35.45	0.8390	0.2542	496.67	32.46
